# The Impact of Age, Sex and Socioeconomic Deprivation on Outcomes in a Colorectal Cancer Screening Programme

**DOI:** 10.1371/journal.pone.0066063

**Published:** 2013-06-12

**Authors:** David Mansouri, Donald C. McMillan, Yasmin Grant, Emilia M. Crighton, Paul G. Horgan

**Affiliations:** 1 Academic Unit of Surgery, School of Medicine-University of Glasgow, Glasgow Royal Infirmary, Glasgow, United Kingdom; 2 Public Health Directorate, NHS Greater Glasgow & Clyde, West House, Gartnaval Royal Hospital, Glasgow, United Kingdom; The Chinese University of Hong Kong, Hong Kong

## Abstract

**Background:**

Population-based colorectal cancer screening has been shown to reduce cancer specific mortality and is used across the UK. Despite evidence that older age, male sex and deprivation are associated with an increased incidence of colorectal cancer, uptake of bowel cancer screening varies across demographic groups. The aim of this study was to assess the impact of age, sex and deprivation on outcomes throughout the screening process.

**Methods:**

A prospectively maintained database, encompassing the first screening round of a faecal occult blood test screening programme in a single geographical area, was analysed.

**Results:**

Overall, 395 096 individuals were invited to screening, 204 139 (52%) participated and 6 079 (3%) tested positive. Of the positive tests, 4 625 (76%) attended for colonoscopy and cancer was detected in 396 individuals (9%). Lower uptake of screening was associated with younger age, male sex and deprivation (all p<0.001). Only deprivation was associated with failure to proceed to colonoscopy following a positive test (p<0.001). Despite higher positivity rates in those that were more deprived (p<0.001), the likelihood of detecting cancer in those attending for colonoscopy was lower (8% most deprived vs 10% least deprived, p = 0.003).

**Conclusion:**

Individuals who are deprived are less likely to participate in screening, less likely to undergo colonoscopy and less likely to have cancer identified as a result of a positive test. Therefore, this study suggests that strategies aimed at improving participation of deprived individuals in colorectal cancer screening should be directed at all stages of the screening process and not just uptake of the test.

## Introduction

Colorectal cancer is the third most common cancer in the Western world and is second only to lung cancer as a cause of cancer death in the combined male and female populations in the UK. Around 40,000 people are diagnosed with bowel cancer each year in the UK alone and around 16,000 deaths occur annually from the disease. Incidence increases with age with over 80% of cases occurring in patients over the age of 60 y. Males in the UK have a lifetime risk of 1 in 14 of contracting the disease and females a risk of 1 in 19 [1]. Individual risk has also been associated with socioeconomic deprivation in particular in males, with those in the least deprived categories having a 20% lower incidence compared with those in the most deprived [2]. There is also evidence that following a diagnosis of colorectal cancer, those who are more socioeconomically deprived have both poorer cancer specific and overall survival [3].

There is good evidence that screening for colorectal cancer using the guaiac-based faecal occult blood test (gFOBt) increases the number of early stage cancers diagnosed (Dukes A and B) and consequently reduces cancer specific mortality [4]–[6]. In addition, there is some evidence that screening may reduce the incidence of bowel cancer through removal of cancer precursors; dysplastic polyps [7]. In response to this evidence, bowel screening programmes have been introduced across the UK and have seen overall participation rates of just over 50% [8], [9]. Within this, however, participation rates may vary widely across demographic groups, with those who are male, younger, more deprived and more ethnically diverse reported less likely to engage in the process [8], [10]. This has added further weight to the suggestion that such individuals may gain a disproportionately low share of the survival benefits from screening [11]–[13].

The Scottish Bowel Screening Programme (SBoSP) was introduced in a staged manner across Scotland beginning in 2007. It is a biennial programme which invites all males and females between the age of 50 and 74 years to take part. Recently this has been extended to allow those over the age of 74 to opt into the programme. This differs from the English screening programme which initially included all individuals aged 60 to 69 and now is currently being extended to include those up to their 75^th^ birthday. The SBoSP was introduced in NHS Greater Glasgow and Clyde (NHS GG&C) in April 2009. In particular, this geographical area is recognised to be one in which there is a high incidence of multiple deprivation. For example, NHS GG&C encompasses an area that includes 49% of the most deprived areas in Scotland. This is the highest proportion of any health board in Scotland and can be compared to the second highest proportion which is 7% in Edinburgh [14].

The aim of the present study was to examine, in an area of multiple deprivation, the impact of age, sex and socioeconomic deprivation not only on uptake, but throughout all stages of the screening process.

## Patients and Methods

Beginning in April 2009 all males and females between the age of 50 and 74 and registered with a General Practitioner (GP) in NHS GG&C were identified via their Community Health Index (CHI) and invited to participate in the SBoSP. Each participant was initially sent a pre-notification letter advising them that they would be receiving an invite to participate in the screening programme. Each participant was then sent a gFOBt kit and asked to provide 2 samples from 3 separate faecal specimens [hema-screen, Immunostics, Ocean, New Jersey, USA, supplied by Alpha Laboratories, Eastleigh, Hampshire, UK]. These were deposited on 6 oval windows provided in the kit and then the kit returned to the Scottish Bowel Screening Centre (Kings Cross Hospital, Dundee) for analysis in a pre-marked foil envelope. Tests were not rehydrated on arrival at the analysis centre and no dietary restrictions were imposed on test subjects. Tests were classified as positive if 5 out of 6 windows were positive, and weakly positive if 1–4 windows were positive. In the case of a weakly positive result or a spoiled gFOBt kit, a further faecal immunochemical test (FIT) kit was sent out [hema-screen SPECIFIC, Immunostics, Ocean, New Jersey, USA, supplied by Alpha Laboratories, Eastleigh, Hampshire, UK] [15]. Following a positive test result, individuals were pre-assessed, either face-to-face or following telephone consultation, by a bowel screening endoscopy nurse and then referred on for colonoscopy if this was deemed suitable. If colonoscopy was unsuccessful then further bowel imaging by barium enema or CT pneumocolonography was attempted. As screening is biennial, two years worth of test invitations was taken to comprise one complete screening round.

Participant details were obtained from a prospectively maintained database held by the Public Health Screening Unit in NHS GG&C. Data on endoscopic findings and pathological diagnosis was obtained retrospectively from clinical information systems by two clinicians (DM and YG). These results formed the basis of the analysis. The presence of uncomplicated diverticulosis and hyperplastic polyps was noted as normal findings. The presence of colitis/proctitis, angiodysplasia, or haemorrhoids were classified as non-neoplastic pathology as a cause of the positive test.

Deprivation category was calculated using the Scottish Index of Multiple Deprivation 2009 (SIMD) which is an index of relative deprivation combining multiple detailed indicators across 7 domains [16]. The overall index is a weighted rank for each of these domains; income (28%), employment (28%), health (14%), education, skills and training (14%), geographic access (9%), crime (5%) and housing (2%). Based on this weighted rank, the 6505 postcodes in Scotland are ranked in order of deprivation. Each postcode represents a small geographical area containing around 750 people. Quintiles of deprivation were used to assign individuals a relative deprivation category based on their postcode at time of colonoscopy with the first quintile representing the most deprived and the fifth quintile, the least deprived. Therefore, those in the first quintile, the most deprived, were likely to have higher levels of poverty, unemployment and poorer health than those in the fifth quintile, who were least deprived.

In those individuals in whom a pathological diagnosis of dysplastic polyps was reached, they were classified as being of a low risk, intermediate risk or high risk of subsequent development of colorectal cancer as per British Society of Gastroenterology (BSG) guidelines [17]. (low risk; 1 to 2 polyps <1 cm: intermediate risk; 3–4 polyps <1 cm or ≥1 polyp≥1 cm: high risk; ≥5 polyps or ≥3 polyps of which ≥1 is ≥1 cm). Low risk polyps were termed non-significant and intermediate or high risk polyps termed significant.

In those individuals in whom a diagnosis of colorectal cancer was reached, initial staging for comparison was following endoscopic and imaging modalities. Subsequent, pathological classification in those who underwent operations was by the standard Turnbull modification of Dukes stage whereby all cases with metastatic disease are classified as Dukes D [18], [19]. Individuals in whom a polyp cancer was considered to be completely excised endoscopically and hence did not undergo further colonic resection, were presumed to be node negative and classified as Dukes A.

The positive predictive value (PPV) for detecting cancer was defined as the number of individuals in whom a cancer was detected divided by the number of individuals undergoing colonoscopy. The PPV for neoplasia was defined as the number of individuals in whom a cancer or dysplastic polyp was identified divided by the number of individuals undergoing colonoscopy and the PPV for significant neoplasia was the number of individuals with either a cancer or significant polyps divided by the number of individuals undergoing colonoscopy. The cancer detection rate was defined as the number of individuals detected with cancer divided by the number who responded to screening test invitation.

### Ethics Statement

Approval for the study was given by the Scottish Bowel Screening Programme in NHS GG&C as a review of service provision, therefore as per National Research and Ethics Service (NRES) guidance no formal ethical review was required and individual patient consent was not required. Data was stored and analysed in an anonymised manner. All research was carried out in the geographical area of study.

### Statistical Analysis

Associations between categorical variables were examined using χ^2^ tests for linear trend unless otherwise specified. Multivariate analysis was carried out using binary logistical regression. A value of p<0.05 was considered statistically significant. Statistical analysis was performed using SPSS software (SPSS Inc., Chicago, IL, USA).

## Results

**Figure 1:**
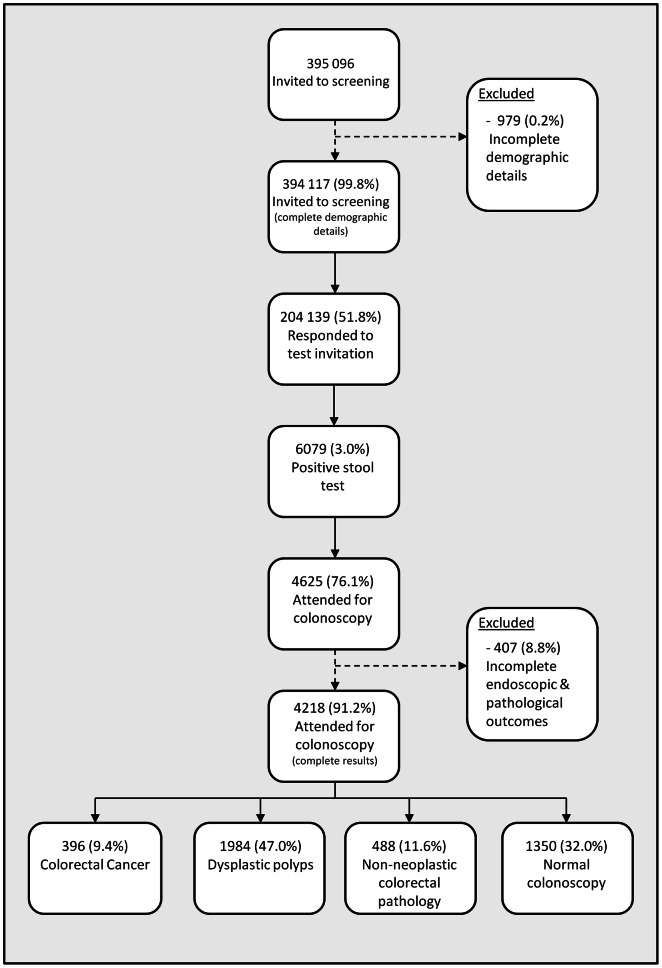
Outcomes of the first round of colorectal cancer screening in NHS GG&C.

From April 2009 to March 2011 inclusive, 395 096 individuals were invited to participate in screening in whom full details on age, sex and deprivation were available for 394 117 (99.8%) which were included for analysis. 192 294 (48.8%) were in the two most deprived quintiles of deprivation and 192 312 (48.9%) were male. The demographic details are shown in Table 1 and a flow diagram of the cohort is outlined in Figure 1.

**Table 1:** Outcome of screening invitation within the SBoSP in NHS GG&C.

	All individuals invited to screening	Responders	Non-responders	p-value	Multivariate analysis	p-value
	n (%)	n (%)	n (%)		O.R. (95% CI)	
	394 117	204 139 (52%)	189 978 (48%)			
**Age**						
**≤55** **y**	135 145 (34%)	61 858 (30%)	73 287 (39%)		1.00	
**56** **y–64** **y**	126 032 (32%)	68 797 (34%)	57 235 (30%)		1.41 (1.39–1.44)	<0.001
**≥65** **y**	132 940 (34%)	73 484 (36%)	59 456 (31%)	<0.001	1.47 (1.45–1.49)	<0.001
**Sex**						
** Male**	192 912 (49%)	92 723 (45%)	100 189 (53%)		1.00	
** Female**	201 205 (51%)	111 416 (55%)	89 789 (47%)	<0.001	1.34 (1.32–1.35)	<0.001
**Deprivation quintile**						
** 1 (most deprived)**	125 263 (32%)	52 604 (26%)	72 659 (38%)		1.00	
** 2**	67 031 (17%)	32 838 (16%)	34 193 (18%)		1.32 (1.30–1.35)	<0.001
** 3**	64 237 (16%)	34 984 (17%)	29 253 (15%)		1.65 (1.62–1.68)	<0.001
** 4**	58 687 (15%)	34 230 (17%)	24 457 (13%)		1.95 (1.91–1.99)	<0.001
** 5 (least deprived)**	78 899 (20%)	49 483 (24%)	29 416 (16%)	<0.001	2.34 (2.29–2.38)	<0.001

### Outcome of Screening Invitation

Of the 394 117 people invited, 204 139 (51.8%) chose to take up the test (Table 1). Uptake was higher in older individuals (45.8% vs 54.6% vs 55.3%, p<0.001), females (55.4% vs 48.1%, p<0.001), and those who were less socioeconomically deprived (62.7% least deprived vs 42.0% most deprived, p<0.001). Due to significant interrelationships between age, sex and deprivation in the cohort invited to screening, multivariate analysis was undertaken. The relationships between age, sex and deprivation identified on univariate analysis remained significant (p<0.001).

### Outcome of Screening Test

Of the 204 139 who took up the test, 6 079 (3.0%) tested positive (Table 2). Positivity rates were higher with advancing age (2.0% vs 2.7% vs 4.1%, p<0.001), in males (3.8% vs 2.3%, p<0.001), and in those who were more deprived (4.2% most deprived vs 1.9% least deprived, p<0.001). Due to a significant interrelationship between age and sex in the cohort responding to the screening invitation, multivariate analysis was undertaken. The relationships with both increasing age and increasing deprivation, and higher positivity rates, remained significant (p<0.001).

**Table 2:** Outcome of screening test within the SBoSP in NHS GG&C.

	All individuals responding to screening invite	Positive screening test	Negative screening test	p-value	Multivariate analysis	p-value
	n (%)	n (%)	n (%)		O.R. (95% CI)	
	204 139	6 079 (3%)	198 060 (97%)			
**Age**						
**≤55** **y**	61 858 (30%)	1 256 (21%)	60 602 (31%)		1.00	
**56** **y–64** **y**	68 797 (34%)	1 842 (30%)	66 955 (34%)		1.35 (1.26–1.45)	<0.001
**≥65** **y**	73 484 (36%)	2 981 (49%)	70 503 (36%)	<0.001	2.07 (1.93–2.21)	<0.001
**Sex**						
** Male**	92 723 (45%)	3 560 (59%)	89 163 (45%)		1.00	
** Female**	111 416 (55%)	2 519 (41%)	108 897 (55%)	<0.001	0.57 (0.54–0.60)	<0.001
**Deprivation quintile**						
** 1 (most deprived)**	52 604 (26%)	2 237 (37%)	50 367 (25%)		1.00	
** 2**	32 838 (16%)	1 137 (19%)	31 701 (16%)		0.80 (0.75–0.86)	<0.001
** 3**	34 984 (17%)	989 (16%)	33 995 (17%)		0.65 (0.60–0.70)	<0.001
** 4**	34 230 (17%)	766 (13%)	33 464 (17%)		0.51 (0.47–0.56)	<0.001
** 5 (least deprived)**	49 483 (24%)	950 (16%)	48 533 (25%)	<0.001	0.44 (0.40–0.47)	<0.001

### Attendance for Colonoscopy

Of the 6 079 positive cases, following pre-assessment, 4 625 (76.1%) individuals attended for colonoscopy (Table 3). Failure to attend for colonoscopy was not associated with age or sex. However, it was associated with deprivation (80.0% least deprived vs 73.3% most deprived, p<0.001).

**Table 3:** Attendance for colonoscopy within the SBoSP in NHS GG&C.

	All individuals with a positive screening test	Attended for colonoscopy	Did not attend for colonoscopy	p-value
	n (%)	n (%)	n (%)	
	6 079	4625 (76%)	1454 (24%)	
**Age**				
**≤55** **y**	1 256 (21%)	961 (21%)	295 (20%)	
**56** **y–64** **y**	1 842 (30%)	1 416 (31%)	426 (29%)	
**≥65** **y**	2 981 (49%)	2 248 (49%)	733 (50%)	0.331
**Sex**				
** Male**	3 560 (59%)	2 732 (59%)	828 (57%)	
** Female**	2 519 (41%)	1 893 (41%)	626 (43%)	0.152
**Deprivation quintile**				
** 1 (most deprived)**	2 237 (37%)	1 639 (35%)	598 (41%)	
** 2**	1 137 (19%)	868 (19%)	269 (19%)	
** 3**	989 (16%)	764 (17%)	225 (16%)	
** 4**	766 (13%)	594 (13%)	172 (12%	
** 5 (least deprived)**	950 (16%)	760 16%)	190 (13%)	<0.001

### Outcome of Colonoscopy

#### Cancer

Of the 4 625 individuals who underwent colonoscopy, full endoscopic and pathological results were available for 4 218 (91.2%) which were included for analysis. Cancer was detected in 396 individuals (9.4%) (Table 4). Increasing age (5.3% vs 8.1% vs 11.9%, p<0.001) and male sex (10.5% vs 7.7%, p = 0.002) were associated with higher PPVs of cancer at colonoscopy. Despite the highest test positivity rates in the most deprived individuals, being less deprived was actually associated with a higher PPV for cancer (10.5% least deprived vs 7.8% most deprived, p = 0.003). Due to significant interrelationships between age, sex and deprivation within those who underwent colonoscopy, multivariate analysis was undertaken. Older age and male sex remained significant (both p<0.05), however the relationship between reduced deprivation and a higher likelihood of cancer remained significant in those in the 3 least deprived quintiles of deprivation only.

**Table 4:** Detection of cancer at colonoscopy within the SBoSP in NHS GG&C.

	All individuals with a colonoscopy result	Cancer	Not cancer	p-value	Multivariate analysis	p-value
	n (%)	n (%)	n (%)		O.R. (95% CI)	
	4 218	396 (9%)	3 822 (91%)			
**Age**						
**≤55** **y**	880 (21%)	47 (12%)	833 (22%)		1.00	
**56** **y–64** **y**	1 289 (31%)	105 (27%)	1 184 (31%)		1.53 (1.08–2.19)	0.018
**≥65** **y**	2 049 (49%)	244 (62%)	1 805 (47%)	<0.001	2.38 (1.72–3.29)	0.001
**Sex**						
** Male**	2 506 (59%)	264 (67%)	2 242 (59%)		1.00	
** Female**	1 712 (41%)	132 (33%)	1 580 (41%)	0.002	0.73 (0.58–0.90)	0.004
**Deprivation quintile**						
** 1 (most deprived)**	1516 (36%)	118 (30%)	1 398 (37%)		1.00	
** 2**	791 (19%)	67 (17%)	724 (19%)		1.06 (0.78–1.45)	0.710
** 3**	676 (16%)	74 (19%)	602 (16%)		1.42 (1.05–1.94)	0.025
** 4**	530 (13%)	63 (16%)	467 (12%)		1.60 (1.15–2.21)	0.005
** 5 (least deprived)**	705 (17%)	74 (19%)	631 (17%)	0.003	1.36 (1.00–1.85)	0.050

Of the 396 individuals with cancer, completing staging information was present in 379 (95.7%). Of these, 181 (48.1%) tumours were Dukes A, 80 (21.1%) were Dukes B, 93 (24.5%) were Dukes C and 25 (6.6%) were Dukes D. There was no effect of age, sex and deprivation on the stage of cancer detected through screening.

#### Dysplastic polyps

Of the 4 218 with colonoscopy results, 1 984 (47.0%) had dysplastic polyps detected (Table 5). Of the 1 984 individuals with dysplastic polyps, 662 (33.4%) individuals had non-significant polyps, and 1322 (66.6%) individuals had significant polyps (937 (70.8%) were intermediate risk and 385 (29.1%) were high risk). This gave a PPV for neoplasia (cancer or polyp) of 56.4% and a PPV for significant neoplasia (significant polyp or cancer) of 40.7% at colonoscopy. Increasing age (43.6% vs 56.3% vs 62.0%, p<0.001∶30.2% vs 40.2% vs 45.6%, p<0.001) and male sex (66.0% vs 42.5%, p<0.001∶48.4% vs 29.5%, p<0.001) were associated with higher PPVs of both of these measures. Again, despite the highest test positivity rate in the most deprived individuals, being less deprived was actually associated with a higher PPV for both neoplasia and significant neoplasia (58.7% least deprived vs 53.3% most deprived, p = 0.016∶45.0% least deprived vs 36.9% most deprived, p<0.001). There was no apparent association between age and deprivation noted, therefore the data was further stratified by sex only. The relationship with increasing age and a higher PPV for neoplasia and significant neoplasia age remained (p<0.001). The relationship between deprivation and lower PPV for both neoplasia and significant neoplasia remained only in males (p<0.005).

**Table 5:** Complete outcomes of colonoscopy within the SBoSP in NHS GG&C.

	All individuals at colonoscopy	Colorectal Cancer	Dysplastic polyps	Non-neoplastic colorectal pathology^1^	Normal colonoscopy	p-value
	n (%)	n (%)	n (%)	n (%)	n (%)	
	4 218	396 (9%)	1 984 (47%)	488 (12%)	1 350(32%)	
**Age**						
**≤55** **y**	880 (21%)	47 (12%)	337 (17%)	131 (27%)	365 (27%)	
**56** **y–64** **y**	1 289 (31%)	105 (27%)	621 (31%)	150 (31%)	413 (31%)	
**≥65** **y**	2 049 (49%)	244 (62%)	1 026 (52%)	207 (42%)	572 (42%)	<0.001
**Sex**						
** Male**	2 506 (59%)	264 (67%)	1 389 (70%)	245 (50%)	608 (45%)	
** Female**	1 712 (41%)	132 (33%)	595 (30%)	243 (50%)	742 (55%)	<0.001
**Deprivation quintile**						
** 1 (most deprived)**	1 516 (36%)	118 (30%)	690 (35%)	179 (37%)	529 (39%)	
** 2**	791 (19%)	67 (17%)	395 (20%)	82 (17%)	247 (18%)	
** 3**	676 (16%)	74 (19%)	319 (16%)	89 (18%)	194 (14%)	
** 4**	530 (13%)	63 (16%)	240 (12%)	56 (12%)	171 (13%)	
** 5 (least deprived)**	705 (17%)	74 (19%)	340 (17%)	82 (17%)	209 (16%)	0.001

1 Includes patients with colitis/proctitis, angiodysplasia and haemorrhoids.

Within the 1 984 individuals with dysplastic polyps, the presence of significant polyps was associated with being male (68.3% vs 62.7%, p = 0.015) and being less deprived (71.5% least deprived vs 63.9% most deprived, p = 0.006) (Table 6). There was no association with age noted (p = 0.452). Within those with significant polyps, there were no significant interrelationships noted between age, sex and socioeconomic deprivation therefore multivariate analysis was not undertaken.

**Table 6:** The effect of age, sex and deprivation on the likelihood of significant polyps at colonoscopy the SBoSP in NHS GG&C.

	All individuals with dysplastic polyps	Significant polyps (intermediate/high-risk)	Non-significant polyps (low-risk)	p-value
	n (%)	n (%)	n (%)	
	1 984 (47%)	1 322 (67%)	662 (33%)	
**Age**				
**≤55** **y**	337 (17%)	219 (17%)	118 (18%)	
**56** **y–64** **y**	621 (31%)	413 (31%)	208 (31%)	
**≥65** **y**	1 026 (52%)	690 (52%)	336 (51%)	0.452
**Sex**				
** Male**	1 389 (70%)	949 (72%)	440 (67%)	
** Female**	595 (30%)	373 (28%)	222 (34%)	0.015
**Deprivation quintile**				
** 1 (most deprived)**	690 (35%)	441 (33%)	249 (38%)	
** 2**	395 (20%)	259 (20%)	136 (21%)	
** 3**	319 (16%)	208 (16%)	111 (17%)	
** 4**	240 (12%)	171 (13%)	69 (10%)	
** 5 (least deprived)**	340 (17%)	243 (18%)	97 (15%)	0.006

#### Non-neoplastic pathology

Of the 4 218 with colonoscopy results, 488 (11.6%) had non-neoplastic colorectal pathology identified as being a cause for the positive test result (Table 5). Younger age (14.9% vs 11.6% vs 10.1%, p<0.001), and being female (14.2% vs 9.8%, p<0.001) was associated with an increased likelihood of non-neoplastic colorectal pathology being identified. No association with deprivation was found (p = 0.935). The data was further stratified by sex and the relationship with younger age and higher likelihood of having non-neoplastic colorectal pathology identified remained significant (p<0.05).

#### Normal colonoscopy

Of the 4 218 with colonoscopy results, 1 350 (32.0%) had a normal colonoscopy (Table 5). Decreasing age (41.5% vs 32.0% vs 27.9%, p<0.001), female sex (43.3% vs 24.3%, p<0.001) and increasing deprivation (34.9% most deprived vs 29.6% least deprived, p = 0.012) were all associated with a higher likelihood of a normal colonoscopy. There was no apparent association between age and deprivation noted, therefore the data was further stratified by sex only. The relationship between younger age and a higher likelihood of a normal colonoscopy remained (p<0.001). No relationship with deprivation was seen in females, and a non-significant trend in males was seen (23.8% least deprived vs 27.4% most deprived, p = 0.099).

### Cancer Detection Rates

The cancer detection rates was 0.19% overall. This was significantly higher in males (0.29% vs 0.12%, p<0.001), older individuals (0.08% vs 0.15% vs 0.33%, p<0.001) and more deprived individuals (0.22% most deprived vs 0.15% least deprived, p = 0.006). There was an apparent association between age and deprivation noted, therefore the data was further stratified by both sex and age groups. The relationship between both increasing age and increasing deprivation, and higher cancer detection rates remained significant (all p<0.05). Converting the cancer detection rate to a number needed to test to identify 1 patient with colorectal cancer yielded an overall value of 515 individuals. This was lower with advancing age (1289 individuals vs 655 individuals vs 301 individuals) and male sex (351 males vs 844 females). The number needed to test was lower with more deprived individuals (446 most deprived vs 669 least deprived).

## Discussion

The results of the present study show that age, sex and socioeconomic deprivation have a significant impact throughout the colorectal cancer screening pathway. Males were less likely to respond to screening, more likely to test positive and more likely to have cancer diagnosed following a positive test. This was also the case with older individuals. Furthermore, those who were more deprived were less likely to respond to screening, more likely to test positive, however, were more likely to fail to proceed to colonoscopy and less likely to have cancer or polyps diagnosed at colonoscopy. Therefore, this study suggests that strategies aimed at improving participation of deprived individuals in colorectal cancer screening should be directed at all stages of the screening process and not just uptake of the test.

Overall, our uptake of the screening test (52%) was slightly below both figures from the first round of the Scottish pilot study and first round of the English screening programme [9], [20]. This is despite individuals in our area being sent a pre-notification letter that has previously been shown to improve participation rates, something that was not present in the other studies [21]. The lower overall uptake may be due to the high level of deprivation in our population when compared to both the Scottish pilot study and the English figures (32% of our population invited to screening were in the most deprived quintile of deprivation compared to 10% in the most deprived quintile in the Scottish pilot study and 20% in the most deprived quintile in the English programme). Within this, a poorer response to invitations in younger individuals, males, and those who were more deprived was also seen. This effect appeared cumulative, for example younger, males who were most deprived had a 34% response rate compared to older, females, who were least deprived who had a response rate of 69%. The gradient of disparity in response to screening invite was largest in the socioeconomically deprived highlighting the important role that deprivation has in determining the uptake of a colorectal cancer programme.

It was also of note that deprivation was the only variable associated with failing to proceed to colonoscopy following a positive result. The reasons for failing to proceed to colonoscopy can either be participant factors (choosing not to participate) or medical factors (participant not being fit enough to proceed). Indeed, overall health is a facet of deprivation and hence more deprived individuals may be less likely to be as fit to undergo a colonoscopy as less deprived individuals. It has already been noted that one of the disadvantages of screening is the anxiety and stress of a positive result in an otherwise asymptomatic individual and in an individual that has a positive screening test and is not fit enough to proceed to colonoscopy, this effect may be magnified [22]. Our study reinforces both recent results from the English screening programme and results from the Scottish pilot study that have shown increased rates of non-attendance in those who are more socioeconomically deprived [13], [23]. As these previous studies did not include those deemed unsuitable for colonoscopy, uptake rates were higher, however it is worth noting that the gradient in disparity associated with deprivation was smaller than the results of the present study. One explanation may be the differing spectrum of deprivation in different geographical areas. It is important that further work focuses on the specific barriers to proceeding to colonoscopy.

The positivity rate (3%), and PPV for cancer at colonoscopy (9%) were similar to previously reported figures from both Scotland and England [9], [20]. However, within this, wide variations throughout the demographics were noted. The higher cancer detection rate found in older, males, who were more deprived by this study was expected, as this is indicative of the overall incidence of the disease [1]. However, it was surprising that there was an inverse relationship between the PPV for cancer at colonoscopy and deprivation. The results of the present study found a higher PPV for cancer at colonoscopy in those who were less deprived. The reasons for this remain unclear. It is thought that not all screen detected cancers are asymptomatic, and that individuals who choose to take up screening are more likely to have lower gastrointestinal symptoms [24]. It has been suggested that rather than only identifying occult disease, screening represents another pathway for symptomatic individuals to choose to present.

Therefore, one plausible explanation is that the lower PPV for cancer at colonoscopy exhibited by those who were more deprived was related to the fact that they had a higher incidence of other non-neoplastic colorectal pathology. While not directly related to cancer detection, had a higher rate of non-neoplastic pathology, such as colitis, be detected in this subpopulation then it may be an added benefit of screening. However, the results of this study do not support this theory. The lower PPV for cancer appeared to be due to a higher number of normal colonoscopies in the more deprived group, which can be viewed as a ‘true’ false positive rate of the test.

False positives with gFOBt can be due to upper gastrointestional (GI) causes or dietary factors, although a link with either of these and socio-economic deprivation has not previously been demonstrated [25]. In a study by Rockey et al. healthy volunteers were given small volumes of their own blood to ingest. gFOBt’s and FIT’s were subsequently examined, with the gFOBt’s found be positive and the FIT’s negative. The positivity rates of gFOBt’s increased with increasing amounts of ingested blood suggesting that a relationship between blood in the upper GI tract and positivity exists [26]. Indeed, there is ongoing debate as to role of upper GI endoscopy in patients who are gFOBt positive and colonoscopy negative [27]. However, the applicability of this to the present study is not clear. A substantial number of patients in the present study will only have been weakly gFOBt positive and will have proceeded to colonoscopy following a subsequent positive FIT. Further work is therefore required to explore the disparity between a higher test positivity rate and a lower PPV of cancer at colonoscopy associated with deprivation within the context of a reflex gFOBt/FIT screening programme.

The PPV for detecting cancer is not the only significant feature of the screening test, as the elimination of pre-cancerous dysplastic polyps is also important to monitor. In fact, a high adenoma pick up rate has been shown to reduce the incidence of colorectal cancer within a screened population, and the removal of dysplastic polyps at colonoscopy has recently been shown to reduce cancer-specific mortality in the long term [5], [28]. The fact that our findings were consistently observed across the PPV for detecting cancer, and both the PPV for neoplasia and significant neoplasia is further validation of the impact of age, sex and deprivation and to date has not been previously reported. Moreover, the present study is able to examine in detail different types of dysplastic polyps. It would be overly simplistic to group all dysplastic polyps as being of equal relevance within a screening programme and the present study has sufficiently large numbers to allow such a subanalysis to take place.

This is a retrospective study using a prospectively maintained database and has a number of limitations. First of all, the proportion of patients in the study who had previously undergone colonoscopy or other lower GI investigation is unknown. This may have affected both an individuals’ attitude towards engaging in the screening process and the likelihood of finding significant pathology at colonoscopy. Indeed, the multicentre UK Flexible Sigmoidoscopy Trial recruited patients aged 55 to 64 years in NHS GG&C up to March 1999 and there may be some crossover between individuals included in the present study and this previous trial [29]. However, the proportion of such individuals is likely to be less than 10%. Furthermore the present study is not able to assess reasons for non-participation or outcomes in those who chose not to participate. Assessing outcomes, such as a subsequent colonoscopy or cancer diagnosis, in non-responders or those who tested negative requires complex data linkage with population based datasets and such information was not available in the present study. In addition, a positive test in the present study actually represents the outcome from three separate screening pathways; strongly positive gFOBt, positive FIT following a weak gFOBt or a positive FIT following a spoiled/untestable gFOBt. There was limited data on the type of positive test for each individual (either gFOBt or FIT) or compliance with FIT in those who tested weakly positive on gFOBt, and therefore this was not able to be included in analysis.

In summary, this data demonstrates that there are wide variations in uptake and outcomes with colorectal cancer screening in its current reflex gFOBt/FIT format associated with age, sex and socioeconomic deprivation. However, deprivation should be highlighted as the only variable that has a consistent impact throughout all stages of the process. Strategies aimed at improving participation of deprived individuals in colorectal cancer screening should be directed at all stages of the screening process and not just uptake of the screening test.
